# Common Information Components Analysis

**DOI:** 10.3390/e23020151

**Published:** 2021-01-26

**Authors:** Erixhen Sula, Michael C. Gastpar

**Affiliations:** School of Computer and Communication Sciences, École Polytechnique Fédérale de Lausanne, CH-1015 Lausanne, Switzerland; michael.gastpar@epfl.ch

**Keywords:** common information, dimensionality reduction, feature extraction, unsupervised, canonical correlation analysis, CCA

## Abstract

Wyner’s common information is a measure that quantifies and assesses the commonality between two random variables. Based on this, we introduce a novel two-step procedure to construct features from data, referred to as Common Information Components Analysis (CICA). The first step can be interpreted as an extraction of Wyner’s common information. The second step is a form of back-projection of the common information onto the original variables, leading to the extracted features. A free parameter γ controls the complexity of the extracted features. We establish that, in the case of Gaussian statistics, CICA precisely reduces to Canonical Correlation Analysis (CCA), where the parameter γ determines the number of CCA components that are extracted. In this sense, we establish a novel rigorous connection between information measures and CCA, and CICA is a strict generalization of the latter. It is shown that CICA has several desirable features, including a natural extension to beyond just two data sets.

## 1. Introduction

Understanding relations between two (or more) sets of variates is key to many tasks in data analysis and beyond. To approach this problem, it is natural to reduce each of the sets of variates separately in such a way that the reduced descriptions fully capture the *commonality* between the two sets, while suppressing aspects that are individual to each of the sets. This permits to understand the relation between the two sets without obfuscation. A popular framework to accomplish this task follows the classical viewpoint of *dimensionality reduction* and is referred to as Canonical Correlation Analysis (CCA) [1]. CCA seeks the best *linear* extraction, i.e., we consider linear projections of the original variates. Via the so-called Kernel trick, this can be extended to cover arbitrary (fixed) function classes.

Wyner’s common information is a well-known and established measure of the dependence of two random variables. Intuitively, it seeks to extract a third random variable such that the two random variables are conditionally independent given the third, but at the same time that the third variable is as compact as possible. Compactness is measured in terms of the mutual information that the third random variable retains about the original two. The resulting optimization problem is not a convex problem (because the constraint set is not a convex set), and therefore, not surprisingly, closed-form solutions are rare. A natural generalization of Wyner’s common information is obtained by replacing the constraint of conditional independence by a limit on the conditional mutual information. If the limit is set equal to zero, we return precisely to the case of conditional independence. Exactly like mutual information, Wyner’s common information and its generalization are endowed with a clear operational meaning. They characterize the fundamental limits of data compression (in the Shannon sense) for a certain network situation.

### 1.1. Related Work

Connections between CCA and Wyner’s common information have been explored in the past. It is well known that, for Gaussian vectors (standard, non-relaxed), Wyner’s common information is attained by all of the CCA components together, see [2]. This has been further interpreted, see, e.g., [3]. Needless to say, having all of the CCA components together essentially amounts to a one-to-one transform of the original data into a new basis. It does not yet capture the idea of feature extraction or dimensionality reduction. To put our work into context, it is only the *relaxation* of Wyner’s common information [4,5] that permits to conceptualize the sequential, one-by-one recovery of the CCA components, and thus, the spirit of dimensionality reduction.

CCA also appears in a number of other problems related to information measures and probabilistic models. For example, in the so-called Gaussian information bottleneck problem, the optimizing solution can be expressed in terms of the CCA components [6], and an interpretation of CCA as a (Gaussian) probabilistic model was presented in [7].

Generalizations of CCA have appeared before in the literature. The most prominent is built around maximal correlation. Here, one seeks arbitrary remappings of the original data in such a way as to maximize their correlation coefficient. This perspective culminates in the well-known *alternating conditional expectation* (ACE) algorithm [8].

Feature extraction and dimensionality reduction have a vast amount of literature attached to them, and it is beyond the scope of the present article to provide a comprehensive overview. In a part of that literature, information measures play a key role. Prominent examples are independent components analysis (ICA) [9] and the information bottleneck [10,11], amongst others. More recently, feature extraction alternations via information theory are presented in [12,13]. In [12], the estimation of Rényi’s quadratic entropy is studied, whereas, in [13], standard information theoretic measures such as Kullback–Leibler divergence are used for fault diagnosis. Other slightly related feature extraction methods that perform dimensionality reduction on a single dataset include [14,15,16,17,18,19,20]. More concretely, in [14], a sparse Support Vector Machine (SVM) approach is used for feature extraction. In [15], feature extraction is performed via regression by using curvilinearity instead of linearity. In [16], compressed sensing is used to extract features when the data have a sparse representation. In [17], an invariant mapping method is invoked to map the high-dimensional data to low-dimensional data that is based on a neighborhood relation. In [18], feature extraction is performed for a partial learning of the geometry of the manifold. In [19], distance correlation measure (a measure with similar properties as the regular Pearson correlation coefficient) is proposed as a new feature extraction method. In [20], kernel principal component analysis is used to perform feature extraction and allow for the extraction of nonlinearities. In [21], feature extraction is done by a robust regression based approach and, in [22], a linear regression approach is used to extract features.

### 1.2. Contributions

The contributions of our work are the following:

- We introduce a novel suit of algorithms, referred to as CICA. These algorithms are characterized by a two-step procedure. In the first step, a relaxation of Wyner’s common information is extracted. The second step can be interpreted as a form of projection of the common information back onto the original data so as to obtain the respective features. A free parameter γ is introduced to control the complexity of the extracted features.
- We establish that, for the special case where the original data are jointly Gaussian, our algorithms precisely extract the CCA components. In this case, the parameter γ determines how many of the CCA components are extracted. In this sense, we establish a new rigorous connection between information measures and CCA.
- We present initial results on how to extend CICA to more than two variates.
- Via a number of paradigmatic examples, we illustrate that, for *discrete data,* CICA gives intuitively pleasing results while other methods, including CCA, do not. This is most pronounced in a simple example with three sources described in Section 7.1.

### 1.3. Notation

A bold capital letter such as X denotes a random vector, and x its realization. The probability distribution function of random variable *X* will be denoted by $p_{X}$ or p(x) depending on the context. A non-bold capital letter such as *K* denotes a (fixed) matrix, and $K^{H}$ its Hermitian transpose. Specifically, $K_{X}$ denotes the covariance matrix of the random vector X. $K_{XY}$ denotes the covariance matrix between random vectors X and Y. Let P be the set of all probability distribution, discrete or continuous depending on the context. Let us denote with $I_{n}$ the identity matrix of dimension n×n and $0_{n}$ the zero matrix of dimension n×n. We denote by $L{\left[f\left(x\right)\right]}_{x}$ the lower convex envelope of f(x) with respect to *x* and for random variables $L{\left[f\left(X\right)\right]}_{p_{X}}$ is the lower convex envelope of f(X) with respect to $p_{X}$. We denote by $h_{b}\left(x\right):=-x\log x-\left(1-x\right)\log1-x$ the binary entropy for 0≤x≤1.

### 1.4. A Simple Example with Synthetic Data

To set the stage and under the guise of an informal problem statement, let us consider a simple example involving synthetic data. Specifically, we consider two-dimensional data, that is, the vectors X and Y are of length 2. The goal is to extract, separately from each of the two, a one-dimensional description in such a way as to extract the commonality between X and Y while suppressing their individual features. For simplicity, in the present artificial example, we will assume that the entries of the vectors only take value in a small finite set, namely, {0,1,2,3}. To illustrate the point, we consider the following special statistical model:

(1) $$\begin{array}{cc} X & =\left(\begin{array}{c} X_{1}\\ X_{2} \end{array}\right)=\left(\begin{array}{c} U⊕X_{2}\\ X_{2} \end{array}\right), \end{array}$$

and

(2) $$\begin{array}{cc} Y & =\left(\begin{array}{c} Y_{1}\\ Y_{2} \end{array}\right)=\left(\begin{array}{c} U⊕Y_{2}\\ Y_{2} \end{array}\right), \end{array}$$

where $U,X_{2},$ and $Y_{2}$ are mutually independent uniform random variables over the set {0,1,2,3} and ⊕ denotes addition modulo 4.

The reason for this special statistical structure is such that it is obvious what should be extracted, namely, X should be reduced to U, and Y should also be reduced to U. This reduces both X and Y to “one-dimensional” descriptions, and these one-dimensional descriptions capture precisely the dependence between X and Y. In this simple example, all the commonality between X and Y is captured by U. More formally, conditioned on U, the vectors X and Y are conditionally independent.

The interesting point of this example is that any *pair* of components of X and Y are *independent* of each other, such as, for example, $X_{1}$ and $Y_{1}.$ Therefore, the joint covariance matrix of the merged vector (X,Y) is a scaled identity matrix. This implies that any method that only uses the covariance matrix as input, including CCA, cannot find any commonalities between X and Y in this example.

**Figure 1:**
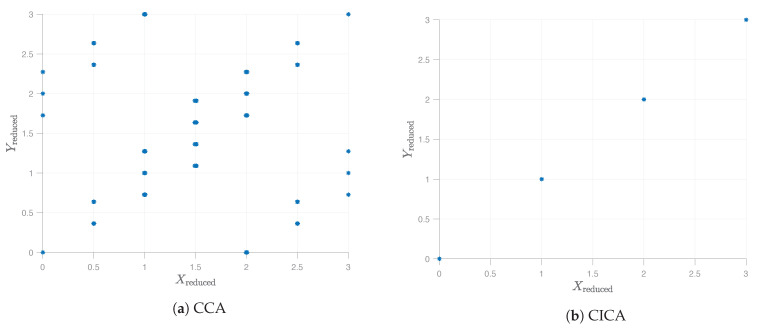
The situation for the synthetic data as described in example described in Section 1.4. Figure 1a shows the scatterplot for two one-dimensional features extracted by CCA. Apparently, the approach is not able to extract the commonality between the vectors X and Y in this synthetic example. Figure 1b shows the performance of the heuristic algorithm of CICA described in Section 6, which, in this simple example, ends up matching the ideal theoretical performance of CICA as in a Generic Procedure 1 for $n\;=\;10^{5}$ data samples.

By contrast, the algorithmic procedure discussed in the present paper will correctly extract the desired answer. In Figure 1, we show numerical simulation outcomes for a couple of approaches. Specifically, in *(a),* we can see that, in this particular example, CCA fails to extract the common features. This, of course, was done on purpose: For the synthetic data at hand, the global covariance matrix is merely a scaled identity matrix, and since CCA’s only input is the covariance matrix, it does not actually do anything in this example. In *(b),* we show the performance of the approximate gradient-descent based implementation of the CICA algorithm proposed in this paper, as detailed in Section 6. In this simple example, this precisely coincides with the ideal theoretical performance of CICA as in a Generic Procedure 1, but, in general, the gradient-descent based implementation is not guaranteed to find the ideal solution.

At this point, we should stress that, for such a simple example, many other approaches would also lead to the same, correct answer. One of them is maximal correlation. In that perspective, one seeks to separately reduce X and Y by applying possibly nonlinear functions f(·) and g(·) in such a way as to maximize the correlation between f(X) and g(Y). Clearly, for the simple example at hand, selecting $f\left(X\right)=X_{1}⊕X_{2}$ and $g\left(Y\right)=Y_{1}⊕Y_{2}$ leads to correlation one, and is thus a maximizer.

Finally, the present example is also too simplistic to express the finer information-theoretic structure of the problem. One step up is the example presented in Section 5 below, where the commonality between X and Y is not merely an equality (the component *U* above), but rather a probabilistic dependency.

## 2. Wyner’s Common Information and Its Relaxation

The main framework and underpinning of the proposed algorithm is Wyner’s common information and its extension, which is briefly reviewed in the sequel, along with its key properties.

### 2.1. Wyner’s Common Information

Wyner’s common information is defined for two random variables *X* and *Y* of arbitrary fixed joint distribution p(x,y).

**Definition** **1** ([23])**.** *For random variables X and Y with joint distribution p(x,y), Wyner’s common information is defined as*

(3) $$\begin{array}{cc} C(X;Y) & =\underset{p(w|x,y)}{\inf}I\left(X,Y;W\right)\;such\;that\;I\left(X;Y|W\right)=0. \end{array}$$

Wyner’s common information satisfies a number of interesting properties. We state some of them below in Lemma 1 for a generalized definition given in Definition 2.

We note that explicit formulas for Wyner’s common information are known only for a small number of special cases. The case of the doubly symmetric binary source is solved completely in [23] and can be written as

(4) $$\begin{array}{cc} C(X;Y) & =1+h_{b}\left(a_{0}\right)-2h_{b}\left(\frac{1-\sqrt{1-2a_{0}}}{2}\right), \end{array}$$

where $a_{0}$ denotes the probability that the two sources are unequal (assuming without loss of generality $a_{0}\leq \frac{1}{2}$). In this case, the optimizing *W* in Equation (3) can be chosen to be binary. Further special cases of discrete-alphabet sources appear in [24].

Moreover, when *X* and *Y* are jointly Gaussian with correlation coefficient ρ, then $C\left(X;Y\right)=\frac{1}{2}\log\frac{1+|\rho |}{1-|\rho |}.$ Note that, for this example, $I\left(X;Y\right)=\frac{1}{2}\log\frac{1}{1-\rho^{2}}.$ This case was solved in [25,26] using a parameterization of conditionally independent distributions, and we have recently found an alternative proof that also extends to the generalization of Wyner’s common information discussed in the next subsection [5].

### 2.2. A Natural Relaxation of Wyner’s Common Information

A natural generalization of Wyner’s common information (Definition 1) is obtained by replacing the constraint of conditional independence with a limit γ on the conditional mutual information, leading to the following:

**Definition** **2** (from [4,5,27])**.** *For random variables X and Y with joint distribution p(x,y), we define*

(5) $$\begin{array}{cc} C_{\gamma }\left(X;Y\right) & =\underset{p(w|x,y)}{\inf}I\left(X,Y;W\right)\;such\;that\;I\left(X;Y|W\right)\leq \gamma . \end{array}$$

This definition appears in slightly different form in Wyner’s original paper (Section 4.2 in [23]), where an auxiliary quantity $\Gamma (\delta_{1},\delta_{2})$ is defined satisfying $C_{\gamma }\left(X;Y\right)=H\left(X,Y\right)-\Gamma \left(0,\gamma \right).$ The above definition first appears in [4]. Comparing Definitions 1 and 2, we see that, for γ=0, we have $C_{0}\left(X;Y\right)=C\left(X;Y\right),$ the regular Wyner’s common information. In this sense, one may refer to $C_{\gamma }\left(X;Y\right)$ as *relaxed* Wyner’s common information.

In line with the discussion following Definition 1, it is not surprising that explicit solutions to the optimization problem in Definition 2 are very rare. In fact, the only presently known general solution concerns the case of jointly Gaussian random variables [5]. The corresponding formula is given below in Theorem 1.

By contrast, the case of the doubly symmetric binary source remains open. An upper bound for this case is given by choosing the auxiliary *W* as

(6) $$\begin{array}{cc} W & =\left\{\begin{array}{cc} X⊕V, & \mathrm{if}X=Y,\\ U, & \mathrm{if}X\neq Y, \end{array}\right. \end{array}$$

where *V* is Bernoulli with probability α and *U* is Bernoulli with probability $\frac{1}{2}$. Thus, the upper bound is

(7) $$\begin{array}{cc} C_{\gamma }\left(X;Y\right) & \leq I\left(X,Y;W\right)=1-\left(1-a_{0}\right)h_{b}\left(\alpha \right)-a_{0}, \end{array}$$

where $\alpha \geq \alpha_{W}$ is chosen such that

(8) $$\begin{array}{cc} I(X;Y|W) & =2h_{b}\left(\left(1-\alpha \right)\left(1-a_{0}\right)+\frac{a_{0}}{2}\right)-\left(1-a_{0}\right)h_{b}\left(\alpha \right)-a_{0}-h_{b}\left(a_{0}\right)=\gamma , \end{array}$$

where

(9) $$\begin{array}{c} \alpha_{W}=\frac{{\left(1-\sqrt{1-2a_{0}}\right)}^{2}}{4(1-a_{0})}. \end{array}$$

Numerical studies (Section 3.5 in [28]) suggest that this upper bound is tight, but no formal proof is available to date.

The following lemma summarizes some basic properties of $C_{\gamma }\left(X;Y\right).$

**Lemma** **1** (partially from [5])**.** *$C_{\gamma }\left(X;Y\right)$ satisfies the following basic properties:*

*1.*  $C_{\gamma }\left(X;Y\right)\geq \max\left\{I\left(X;Y\right)-\gamma ,0\right\}.$
*2.*  Data processing inequality: If X−Y−Z forms a Markov chain,
  then $C_{\gamma }\left(X;Z\right)\leq \min\left\{C_{\gamma }\left(X;Y\right),C_{\gamma }\left(Y;Z\right)\right\}.$
*3.*  $C_{\gamma }\left(X;Y\right)$ is a convex and continuous function of γ for γ≥0.
*4.*  *Tensorization: For n independent pairs ${\left\{\left(X_{i},Y_{i}\right)\right\}}_{i=1}^{n},$ we have that*
  $$C_{\gamma }\left(X^{n};Y^{n}\right)=\min\sum_{i=1}^{n}C_{\gamma_{i}}\left(X_{i};Y_{i}\right),$$
  where the *min* is over all non-negative ${\left\{\gamma_{i}\right\}}_{i=1}^{n}$ satisfying $\sum_{i=1}^{n}\gamma_{i}=\gamma .$
*5.*  If Z−X−Y forms a Markov chain, then $C_{\gamma }\left(\left(X,Z\right);Y\right)=C_{\gamma }\left(X;Y\right).$
*6.*  The cardinality of W may be restricted to |W|≤|X||Y|+1.
*7.*  If f(·) and g(·) are one-to-one functions, then $C_{\gamma }\left(f\left(X\right);g\left(Y\right)\right)=C_{\gamma }\left(X;Y\right).$
*8.*  For discrete X, we have $C_{\gamma }\left(X;X\right)=\max\left\{H\left(X\right)-\gamma ,0\right\}.$

Proofs of items 1–4 are given in [5], and the proofs of items 5–8 are given in Appendix A.

### 2.3. The Non-Convexity of the Relaxed Wyner’s Common Information Problem

It is important to observe that the optimization problem of Definition 2 is not a convex problem. First, we observe that I(X,Y;W) is indeed a convex function of p(w|x,y), which is a well-known fact, see, e.g., (Theorem 2.7.4 in [29]). The issue is with the constraint set. The set of distributions p(w|x,y) for which I(X;Y|W)≤γ is not a convex set. To provide some intuition for the structure of this set, let us consider I(X;Y|W) as a function of p(w|x,y), and examine its (non-)convexity. The relation between the two is described by the epigraph

(10) $$\begin{array}{c} \mathrm{epigraph}\{I(X;Y|W)\}=\{(p(w|x,y),\gamma ):p(w|x,y)\in P,\gamma \geq I(X;Y|W)\}. \end{array}$$

The function I(X;Y|W) is convex in p(w|x,y) if and only if its epigraph is a convex set which would imply that the set of distributions p(w|x,y) for which I(X;Y|W)≤γ is also convex. Now, we present an example that I(X;Y|W) is not a convex function of p(w|x,y).

**Example** **1.** *Let the distributions $p\left(x,y\right),p_{1}\left(w|x,y\right),p_{2}\left(w|x,y\right)$ be*

(11) $$\begin{array}{c} p\left(x,y\right)=\left[\begin{array}{cc} \frac{2}{5} & \frac{1}{10}\\ \frac{1}{10} & \frac{2}{5} \end{array}\right],p_{1}\left(w|x,y\right)=\left[\begin{array}{cccc} \frac{1}{4} & \frac{1}{4} & \frac{1}{4} & \frac{1}{4}\\ \frac{3}{4} & \frac{3}{4} & \frac{3}{4} & \frac{3}{4} \end{array}\right],p_{2}\left(w|x,y\right)=\left[\begin{array}{cccc} \frac{1}{2} & \frac{3}{4} & \frac{3}{4} & \frac{3}{4}\\ \frac{1}{2} & \frac{1}{4} & \frac{1}{4} & \frac{1}{4} \end{array}\right], \end{array}$$

*respectively. For this example, one can evaluate numerically that, under $p_{1}\left(w|x,y\right),$ we have $I_{p_{1}}\left(X;Y|W\right)<0.279$ and under $p_{2}\left(w|x,y\right),$ we have $I_{p_{2}}\left(X;Y|W\right)<0.262$. By the same token, one can show that, under $(p_{1}\left(w|x,y\right)+p_{2}\left(w|x,y\right))/2,$ we have $I_{(p_{1}+p_{2})/2}\left(X;Y|W\right)>0.274$. Hence, we conclude that, for this example,*

(12) $$\begin{array}{cc} I_{(p_{1}+p_{2})/2}\left(X;Y|W\right) & >\frac{1}{2}\left(I_{p_{1}}\left(X;Y|W\right)+I_{p_{2}}\left(X;Y|W\right)\right), \end{array}$$

*which proves that I(X;Y|W) cannot be convex.*

### 2.4. The Operational Significance of the Relaxed Wyner’s Common Information Problem

It is important to note that Wyner’s common information has clear and well-defined operational significance. This is perhaps not central to the detailed explanations and examples given in the sequel. However, it has a role in the appreciation of the rigorous connection established in our work. An excellent description of the operational significance is given in (Section I in [23]), where two separate aspects are identified. The first concerns a source coding scenario with a single encoder and two decoders, one interested in *X* and the other in Y. Three bit streams are constructed by the encoder: One public (to both decoders), and two private streams, one for each decoder. Then, C(X;Y) characterizes the minimum number of bits that must be sent on the public stream such that the total number of bits sent stays at the global minimum, which is well known to be the joint entropy of *X* and Y. If the rate on the public bit stream dips below C(X;Y), it is no longer possible to keep the total rate at the joint entropy. Rather, there is a strict penalty now, and this penalty can be expressed via $C_{\gamma }\left(X;Y\right).$ The second rigorous operational significance concerns the distributed generation of correlated randomness. We have two separate processors, one generating *X* and the other Y. For a fixed desired resulting probability distribution p(x,y), how many common random bits (shared between both processors) are required? Again, the answer is precisely C(X;Y). A connection between caching and the Gray–Wyner network is developed in [30].

## 3. The Algorithm

The main technical result of this paper is to establish that the outcome of a specific procedure induced by the relaxed Wyner’s common information is tantamount to CCA whenever the original underlying distribution is Gaussian. In preparation for this, in this section, we present the proposed algorithm. In doing so, we will assume that the distribution of the data are p(x,y). In many applications involving CCA, the data distributions may not be known, but, rather, a number of samples of X and Y are provided, based on which CCA would then estimate the covariance matrix. A similar perspective can be taken on our procedure, but is left for future work. A short discussion can be found in Section 8 below.

### 3.1. High-Level Description

The proposed algorithm takes as input the distribution p(x,y) of the data, as well as a level γ. The level γ is a non-negative real number and may be thought of as a resolution level or a measure of coarseness: If γ=0, then the full commonality (or common information) between X and Y is extracted in the sense that it is conditioned on the common information, X and Y are conditionally independent. Conversely, if γ is large, then only the most important part of the commonality is extracted. Fixing the level γ, the idea of the proposed algorithm is to evaluate the relaxed Wyner’s Common Information of Equation (5) between the information sources (data sets) at the chosen level γ. This evaluation will come with an associated conditional distribution $p_{\gamma }\left(w|x,y\right),$ namely, the conditional distribution attaining the minimum in the optimization problem of Equation (5). The second half of the proposed algorithm consists of leveraging the minimizing $p_{\gamma }\left(w|x,y\right)$ in such a way as to separately reduce X and Y to those features that best express the commonality. This may be thought of as a type of projection of the minimizing random variable *W* back onto X and Y, respectively. For the case of Gaussian statistics, this can be made precise.

### 3.2. Main Steps of the Algorithm

The algorithm proposed here starts from the joint distribution of the data, p(x,y). Estimates of this distribution can be obtained from data samples $X^{n}$ and $Y^{n}$ via standard techniques. The main steps of the procedure can then be described as follows:

**Generic Procedure 1**  (CICA)**.**

*1.*  Select a real number γ, where 0≤γ≤I(X;Y). This is the compression level: A low value of γ represents low compression, and, thus, many components are retained. A high value of γ represents high compression, and, thus, only a small number of components are retained.
*2.*  *Solve the relaxed Wyner’s common information problem,*
  (13) $$\begin{array}{c} \min_{p(w|x,y)}I\left(X,Y;W\right)\;such\;that\;I\left(X;Y|W\right)\leq \gamma , \end{array}$$
  leading to an associated conditional distribution $p_{\gamma }\left(w|x,y\right).$
*3.*  Using the conditional distribution $p_{\gamma }\left(w|x,y\right)$ found in Step 2), the dimension-reduced data sets can now be found via one of the following three variants:
  *(a)*  *Version 1: MAP (maximum a posteriori):*
    (14) $$\begin{array}{cc} u(x) & =\arg\max_{w}p_{\gamma }\left(w|x\right), \end{array}$$
    (15) $$\begin{array}{cc} v(y) & =\arg\max_{w}p_{\gamma }\left(w|y\right). \end{array}$$
  *(b)*  *Version 2: Conditional Expectation:*
    (16) $$\begin{array}{cc} u(x) & =E[W|X=x], \end{array}$$
    (17) $$\begin{array}{cc} v(y) & =E[W|Y=y]. \end{array}$$
  *(c)*  *Version 3: Marginal Integration:*
    (18) $$\begin{array}{cc} u(x) & =\int_{y}p\left(y\right)E\left[W|X=x,Y=y\right]dy, \end{array}$$
    (19) $$\begin{array}{cc} v(y) & =\int_{x}p\left(x\right)E\left[W|X=x,Y=y\right]dx. \end{array}$$

The present paper focuses on the three versions given here because, for these three versions, we can establish Theorem 2, showing that, in the case of Gaussian statistics, all three versions lead exactly to CCA. Second, we note that, for concrete examples, it is often evident which of the versions is preferable. For example, in Section 5, we consider a binary example where the associated *W* in Step 2 of our algorithm is also binary. In this case, Version 1 will reduce the original binary vector X to a binary scalar, which is perhaps the most desirable outcome. By contrast, Versions 2 and 3 require an explicit embedding of the binary example in the reals, and will reduce the original binary vector X to a real-valued scalar, which might not be as insightful.

## 4. For Gaussian, CICA Is CCA

In this section, we consider the special case where X and Y are jointly Gaussian random vectors. Since the mean has no bearing on either CCA or Wyner’s common information, we will assume it to be zero in the sequel, without loss of generality. One key ingredient for this argument is a well-known change of basis, see, for example [2], which we will now introduce in detail. Note that the mean will not change any mutual information term, thus we assume it to be zero without a loss of generality. We first need to introduce notation for CCA. To this end, let us express the covariance matrices, as usual, in terms of their eigendecompositions as

(20) $$\begin{array}{c} K_{X}=Q_{x}\left(\begin{array}{cc} \Lambda_{r_{X}} & 0\\ 0 & 0_{n-r_{X}} \end{array}\right)Q_{x}^{T} \end{array}$$

and

(21) $$\begin{array}{c} K_{Y}=Q_{y}\left(\begin{array}{cc} \Lambda_{r_{Y}} & 0\\ 0 & 0_{n-r_{Y}} \end{array}\right)Q_{y}^{T}, \end{array}$$

where $r_{X}$ and $r_{Y}$ denote the rank of $K_{X}$ and $K_{Y},$ respectively. Starting from this, we define the matrices

(22) $$\begin{array}{c} K_{X}^{-1/2}=Q_{x}\left(\begin{array}{cc} \Lambda_{r_{X}}^{-1/2} & 0\\ 0 & 0_{n-r_{X}} \end{array}\right)Q_{x}^{T} \end{array}$$

and

(23) $$\begin{array}{c} K_{Y}^{-1/2}=Q_{y}\left(\begin{array}{cc} \Lambda_{r_{Y}}^{-1/2} & 0\\ 0 & 0_{n-r_{Y}} \end{array}\right)Q_{y}^{T}, \end{array}$$

where, for a diagonal matrix Λ with strictly positive entries, $\Lambda_{r_{Y}}^{-1/2}$ denotes the diagonal matrix whose diagonal entries are the reciprocals of the square roots of the entries of the matrix Λ. Using these matrices, the key step is to apply the change of basis

(24) $$\begin{array}{cc} \hat{X} & =K_{X}^{-1/2}X \end{array}$$

(25) $$\begin{array}{cc} \hat{Y} & =K_{Y}^{-1/2}Y. \end{array}$$

In the new coordinates, the covariance matrices of $\hat{X}$ and $\hat{Y},$ respectively, can be shown to be

(26) $$\begin{array}{c} K_{\hat{X}}=\left(\begin{array}{cc} I_{r_{X}} & 0\\ 0 & 0_{n-r_{X}} \end{array}\right) \end{array}$$

and

(27) $$\begin{array}{c} K_{\hat{Y}}=\left(\begin{array}{cc} I_{r_{Y}} & 0\\ 0 & 0_{n-r_{Y}} \end{array}\right). \end{array}$$

Moreover, we have

(28) $$\begin{array}{c} K_{\hat{X}\hat{Y}}=K_{X}^{-1/2}K_{XY}K_{Y}^{-1/2}. \end{array}$$

Let us denote the singular value decomposition of this matrix by

(29) $$\begin{array}{cc} K_{\hat{X}\hat{Y}} & =U\Sigma V^{H}. \end{array}$$

where Σ contains, on its diagonal, the ordered singular values of this matrix, denoted by $\rho_{1}\geq \rho_{2}\geq \ldots \geq \rho_{n}.$ In addition, let us define

(30) $$\begin{array}{cc} \tilde{X} & =U^{H}\hat{X} \end{array}$$

(31) $$\begin{array}{cc} \tilde{Y} & =V^{H}\hat{Y}, \end{array}$$

which implies that $K_{\tilde{X}}=K_{\hat{X}},$$K_{\tilde{Y}}=K_{\hat{Y}},$ and $K_{\tilde{X}\tilde{Y}}=\Sigma$.

Next, we will leverage this change of basis to establish Wyner’s common information and its relaxation for the Gaussian vector case, and then to prove the connection between Generic Procedure 1 and CCA.

### 4.1. Wyner’s Common Information and Its Relaxation in the Gaussian Case

For the case where X and Y are jointly Gaussian random vectors, a full and explicit solution to the optimization problem of Equation (5) is found in [5]. To give some high-level intuition, the proof starts by mapping from X to $\tilde{X}$ and from Y to $\tilde{Y},$ as in Equations (30) and (31). This preserves all mutual information expressions as well as joint Gaussianity. Moreover, due to the structure of the covariance matrices of the vectors $\tilde{X}$ and $\tilde{Y},$ we have that ${\left\{\left({\tilde{X}}_{i},{\tilde{Y}}_{i}\right)\right\}}_{i=1}^{n}$ are *n* independent pairs of Gaussian random variables. Thus, by the tensorization property (see Lemma 1), the vector problem can be reduced to *n* parallel scalar problems. The solution of the scalar problem is the main technical contribution of [5], and we refer to that paper for the detailed proof. The resulting formula can be expressed as in the following theorem.

**Theorem** **1** (from [5])**.** *Let X and Y be jointly Gaussian random vectors of length n and covariance matrix $K_{\left(X,Y\right)}$. Then,*

(32) $$\begin{array}{cc} \; & C_{\gamma }\left(X;Y\right)=\min_{\gamma_{i}:\sum_{i=1}^{n}\gamma_{i}=\gamma }\sum_{i=1}^{n}C_{\gamma_{i}}\left(X_{i};Y_{i}\right), \end{array}$$

*where*

(33) $$\begin{array}{c} C_{\gamma_{i}}\left(X_{i};Y_{i}\right)=\frac{1}{2}\log^{+}\frac{\left(1+\rho_{i}\right)\left(1-\sqrt{1-e^{-2\gamma_{i}}}\right)}{\left(1-\rho_{i}\right)\left(1+\sqrt{1-e^{-2\gamma_{i}}}\right)} \end{array}$$

*and $\rho_{i}$ (for i=1,⋯,n) are the singular values of $K_{X}^{-1/2}K_{XY}K_{Y}^{-1/2},$ where $K_{X}^{-1/2}$ and $K_{Y}^{-1/2}$ are defined to mean that only the positive eigenvalues are inverted.*

As pointed out above, we refer to (Theorem 7 in [5]) for a rigorous proof of this theorem.

### 4.2. CICA in the Gaussian Case and the Exact Connection with CCA

In this section, we consider the proposed CICA algorithm in the special case where the data distribution is p(x,y), a (multivariate) Gaussian distribution. We establish that, in this case, the classic CCA is a solution to all versions of the proposed CICA algorithm. In this sense, CICA is a strict generalization of CCA. CCA is briefly reviewed in Appendix B. Leveraging the matrices *U* and *V* defined via the singular value decomposition in Equation (29), CCA performs the dimensonality reduction

(34) $$\begin{array}{cc} u(x) & =U_{k}^{H}\hat{x}=U_{k}^{H}K_{X}^{-1/2}x \end{array}$$

(35) $$\begin{array}{cc} v(y) & =V_{k}^{H}\hat{y}=V_{k}^{H}K_{Y}^{-1/2}y, \end{array}$$

where the matrix $U_{k}$ contains the first *k* columns of *U* (that is, the *k* left singular vectors corresponding to the largest singular values), and the matrix $V_{k}$ the respective right singular vectors. We refer to these as the “top *k* CCA components.”

**Theorem** **2.** *Let X and Y be jointly Gaussian random vectors. Then:*

*1.*  *The top k CCA components are a solution to* **all three** *versions of Generic Procedure 1.*
*2.*  *The parameter γ controls the number k as follows:*
  (36) $$\begin{array}{cc} k(\gamma ) & =\left\{\begin{array}{cc} n, & if\;0\leq \gamma <ng(\rho_{n}),\\ n-1, & if\;ng\left(\rho_{n}\right)\leq \gamma <\left(n-1\right)g\left(\rho_{n-1}\right)+g\left(\rho_{n}\right),\\ n-2, & if\;\left(n-1\right)g\left(\rho_{n-1}\right)+g\left(\rho_{n}\right)\leq \gamma \\ \; & \;\;\;\;\;\;\;\;<\left(n-2\right)g\left(\rho_{n-2}\right)+g\left(\rho_{n-1}\right)+g\left(\rho_{n}\right),\\ \vdots , & \vdots ,\\ \ell & if\;\left(\ell +1\right)g\left(\rho_{\ell +1}\right)+\sum_{i=\ell +2}^{n}g\left(\rho_{i}\right)\leq \gamma \\ \; & \;\;\;\;\;\;\;\;<\ell g\left(\rho_{\ell }\right)+\sum_{i=\ell +1}^{n}g\left(\rho_{i}\right),\\ \vdots , & \vdots ,\\ 1, & if\;2g\left(\rho_{2}\right)+\sum_{i=2}^{n}g\left(\rho_{i}\right)\leq \gamma <\sum_{i=1}^{n}g\left(\rho_{i}\right),\\ 0, & if\;\sum_{i=1}^{n}g\left(\rho_{i}\right)\leq \gamma , \end{array}\right. \end{array}$$
*where $g\left(\rho \right)=\frac{1}{2}\log\frac{1}{1-\rho^{2}}.$*

**Figure 2:**
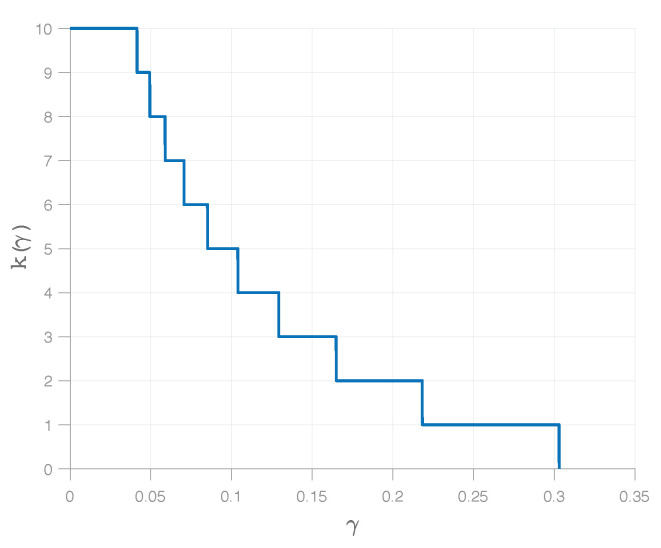
Illustration of the function k(γ) from Theorem 2 for the concrete case where X and Y have n=10 components each and the correlation coefficients are $\rho_{m}=1/\left(m+1\right).$

**Remark** **1.** *Note that k(γ) is a decreasing, integer-valued function. An illustration for a special case is given in Figure 2.*

**Proof.**  The main contribution of the theorem is the first item, *i.e.,* the connection between CCA and Generic Procedure 1 in the case where X and Y are jointly Gaussian. The proof follows along the steps of the CICA procedure: We first show that, in Step 2, when X and Y are jointly Gaussian, then the minimizing *W* may be taken jointly Gaussian with X and Y. Then, we establish that, in Step 3, with the *W* from Step 2, we indeed obtain that the dimension-reduced representations u(x) and v(y) turn into the top *k* CCA components. In detail:

*Step 2 of Generic Procedure 1:* The technical heavy lifting for this step in the case where p(x,y) is a multivariate Gaussian distribution is presented in [5]. We shall briefly summarize it here. In the case of Gaussian vectors, the solution to the optimization problem in Equation (5) is most easily described in two steps. First, we apply the change of basis indicated in Equations (24) and (25). This is a one-to-one transform, leaving all information expressions in Equation (5) unchanged. In the new basis, we have *n* independent pairs. By the tensorization property (see Lemma 1), when X and Y consist of independent pairs, the solution to the optimization problem in Equation (5) can be reduced to *n* separate scalar optimizations. The remaining crux then is solving the scalar Gaussian version of the optimization problem in Equation (5). This is done in (Theorem 3 in [5]) via an argument of factorization of convex envelope. The full solution to the optimization problem is given in Equations (32) and (33). The remaining allocation problem over the non-negative numbers $\gamma_{i}$ can be shown to lead to a water-filling solution, given in (Theorem 8 in [5]). More explicitly, to understand this solution, start by setting γ=I(X;Y). Then, the corresponding $C_{\gamma }\left(X;Y\right)=0$ and the optimizing distribution $p_{\gamma }\left(w|x,y\right)$ trivializes. Now, as we lower γ, the various terms in the sum in Equation (32) start to become non-zero, starting with the term with the largest correlation coefficient $\rho_{1}.$ Hence, an optimizing distribution $p_{\gamma }\left(w|x,y\right)$ can be expressed as $W_{\gamma }=U_{k}^{H}K_{X}^{-1/2}X+V_{k}^{H}K_{Y}^{-1/2}Y+Z,$ where the matrices $U_{k}$ and $V_{k}$ are precisely the top *k* CCA components (see Equations (34) and (35) and the following discussion), and Z is additive Gaussian noise with mean zero, independent of X and Y.

*Step 3 of Generic Procedure 1:* For the algorithm, we need the corresponding conditional marginals, $p_{\gamma }\left(w|x\right)$ and $p_{\gamma }\left(w|y\right).$ By symmetry, it suffices to prove one formula. Changing basis as in Equations (24) and (25), we can write

(37) $$\begin{array}{cc} E[W|X] & =E[U_{k}^{H}\hat{X}+V_{k}^{H}\hat{Y}+Z|\hat{X}] \end{array}$$

(38) $$\begin{array}{cc} \; & =U_{k}^{H}\hat{X}+V_{k}^{H}E\left[\hat{Y}|\hat{X}\right] \end{array}$$

(39) $$\begin{array}{cc} \; & =U_{k}^{H}\hat{X}+V_{k}^{H}\left(E\left[\hat{Y}{\hat{X}}^{H}\right]{\left(E[\hat{X}{\hat{X}}^{H}]\right)}^{-1}\hat{X}\right) \end{array}$$

(40) $$\begin{array}{cc} \; & =U_{k}^{H}\hat{X}+V_{k}^{H}K_{\hat{Y}\hat{X}}\hat{X} \end{array}$$

(41) $$\begin{array}{cc} \; & =U_{k}^{H}\hat{X}+{\left(K_{\hat{X}\hat{Y}}V_{k}\right)}^{H}\hat{X}. \end{array}$$

The first summand contains exactly the top *k* CCA components extracted from X, which is the claimed result. The second summand requires further scrutiny. To proceed, we observe that, for CCA, the projection vectors obey the relationship (see Equation (A12))

(42) $$\begin{array}{cc} u & =\alpha K_{\hat{X}\hat{Y}}v, \end{array}$$

for some real-valued constant α. Thus, combining the top *k* CCA components, we can write

(43) $$\begin{array}{cc} U_{k} & =DK_{\hat{X}\hat{Y}}V_{k}, \end{array}$$

where *D* is a diagonal matrix. Hence,

(44) $$\begin{array}{cc} E[W|X] & =U_{k}^{H}\hat{X}+D^{-1}U_{k}^{H}\hat{X} \end{array}$$

(45) $$\begin{array}{cc} \; & =\tilde{D}U_{k}^{H}\hat{X}, \end{array}$$

where $\tilde{D}$ is the diagonal matrix

(46) $$\begin{array}{cc} \tilde{D} & =I+D^{-1}. \end{array}$$

This is precisely the top *k* CCA components (note that the solution to the CCA problem (A7) is only specified up to a scaling). This establishes the theorem for the case of Version 2) of the proposed algorithm. Clearly, it also establishes that $p_{\gamma }\left(w|x\right)$ is a Gaussian distribution with mean given by (45), thus establishing the theorem for Version 1) of the proposed algorithm. The proof for Version 3 follows along similar lines and is thus omitted. □

## 5. A Binary Example

In this section, we carry through a theoretical study of a somewhat more general case of the example discussed in Section 1.4 that is believed to be within the reach of practical data. In order to do a theoretical study, we need to constrain the data into binary for the reason that computing the Wyner’s common information for doubly binary symmetric source is already known.

Let us illustrate the proposed algorithm via a simple example. Consider the vector $\left(U,X_{2},V,Y_{2}\right)$ of binary random variables. Suppose that (U,V) is a doubly symmetric binary source. This means that *U* is uniform and *V* is the result of passing *U* through a binary symmetric (“bit-flipping”) channel with flip probability denoted by $a_{0}$ to match the notation in (Section 3 in [23]). Without loss of generality, we may assume $a_{0}\leq \frac{1}{2}.$ Meanwhile, $X_{2}$ and $Y_{2}$ are independent binary uniform random variables, also independent of the pair (U,V). We will then form the vectors X and Y as

(47) $$\begin{array}{cc} X & =\left(\begin{array}{c} X_{1}\\ X_{2} \end{array}\right)=\left(\begin{array}{c} U⊕X_{2}\\ X_{2} \end{array}\right), \end{array}$$

and

(48) $$\begin{array}{cc} Y & =\left(\begin{array}{c} Y_{1}\\ Y_{2} \end{array}\right)=\left(\begin{array}{c} V⊕Y_{2}\\ Y_{2} \end{array}\right), \end{array}$$

where ⊕ denotes the modulo-reduced addition, as usual. How do various techniques perform for this example?

- Let us first analyze the behavior and outcome of CCA in this particular example. The key observation is that any pair, amongst the four entries in these two vectors, $X_{1},X_{2},Y_{1},$ and $Y_{2},$ are (pairwise) independent binary uniform random variables. Hence, the overall covariance matrix of the merged random vector ${\left(X^{T},Y^{T}\right)}^{T}$ is merely a scaled identity matrix. This, in turn, implies that CCA as described in Equations (34) and (35) merely boils down to the identity mapping. Concretely, this means that, for CCA, in this example, the best one-dimensional projections are ex aequo any pair of one coordinate of the vector X with one coordinate of the vector Y. As we have already explain above, any such pair is merely a pair of independent (and identically distributed) random variables, so CCA does not extract any dependence between X and Y at all. Of course, this is the main point of the present example.
- How does CICA perform in this example? We selected this example because it represents one of the only cases for which a closed-form solution to the optimization problem in Equation (13) is known, at least in the case γ=0. To see this, let us first observe that, in our example, we have
  (49) $$\begin{array}{c} p\left(u,v,x_{2},y_{2}\right)=p\left(u,v\right)p\left(x_{2}\right)p\left(y_{2}\right). \end{array}$$
  Next, we observe that
  (50) $$\begin{array}{cc} C_{\gamma }\left(X;Y\right) & =C_{\gamma }\left(U,X_{2};V,Y_{2}\right) \end{array}$$
  (51) $$\begin{array}{cc} \; & =C_{\gamma }\left(U;V,Y_{2}\right) \end{array}$$
  (52) $$\begin{array}{cc} \; & =C_{\gamma }\left(U;V\right) \end{array}$$
  where (51) follows from Lemma 1, Item 5, and the Markov chain $X_{2}-U-\left(V,Y_{2}\right)$ that is satisfied from (49). The last Equation (52) follows from Lemma 1, Item 5, and the Markov chain $Y_{2}-V-U$ that is satisfied from (49). That is, in this simple example, solving the optimization problem of Equation (13) is tantamount to solving the optimization problem in Equation (52). For the latter, the solution is well known, see (Section 3 in [23]). Specifically, we can express the conditional distribution $p_{\gamma }\left(w|x,y\right)$ that solves the optimization problem of Equation (13) and is required for Step 3 of Generic Procedure 1 as follows:
  (53) $$\begin{array}{cc} p_{\gamma =0}\left(w|x,y\right) & =\left\{\begin{array}{cc} 1-\nu , & \mathrm{if}\;w=0,x_{1}⊕x_{2}=0,y_{1}⊕y_{2}=0,\\ \nu , & \mathrm{if}\;w=1,x_{1}⊕x_{2}=0,y_{1}⊕y_{2}=0,\\ \nu , & \mathrm{if}\;w=0,x_{1}⊕x_{2}=1,y_{1}⊕y_{2}=1,\\ 1-\nu , & \mathrm{if}\;w=1,x_{1}⊕x_{2}=1,y_{1}⊕y_{2}=1,\\ \frac{1}{2}, & \mathrm{otherwise}. \end{array}\right. \end{array}$$
  where
  (54) $$\begin{array}{cc} \nu & =\frac{1}{2}-\frac{\sqrt{1-2a_{0}}}{2(1-a_{0})}. \end{array}$$
  Let us now apply Version 1 (the MAP version) of Generic Procedure 1. To this end, we also need to calculate $p_{\gamma }\left(w|x\right)$ and $p_{\gamma }\left(w|y\right).$ Again, for γ=0, these can be expressed in a closed form as follows:
  (55) $$\begin{array}{cc} p_{\gamma =0}\left(w|x\right) & =\left\{\begin{array}{cc} 1-a_{1}, & \mathrm{if}\;w=0,x_{1}⊕x_{2}=0,\\ a_{1}, & \mathrm{if}\;w=1,x_{1}⊕x_{2}=0,\\ a_{1}, & \mathrm{if}\;w=0,x_{1}⊕x_{2}=1,\\ 1-a_{1}, & \mathrm{if}\;w=1,x_{1}⊕x_{2}=1, \end{array}\right. \end{array}$$
  where
  (56) $$\begin{array}{cc} a_{1} & =\frac{1}{2}\left(1-\sqrt{1-2a_{0}}\right). \end{array}$$
  The formula for $p_{\gamma }\left(w|y\right)$ follows by symmetry and shall be omitted. The final step is to follow Equations (14) and (15) and find $\arg\max_{w}p_{\gamma =0}\left(w|x\right)$ for each x as well as $\arg\max_{w}p_{\gamma =0}\left(w|y\right)$ for each y. For the example at hand, these can be compactly expressed as
  (57) $$\begin{array}{cc} u(x) & =\arg\max_{w}p_{\gamma }\left(w|x\right)=x_{1}⊕x_{2}=u, \end{array}$$
  (58) $$\begin{array}{cc} v(y) & =\arg\max_{w}p_{\gamma }\left(w|y\right)=y_{1}⊕y_{2}=v, \end{array}$$
  from the fact that $0\leq a_{0}\leq \frac{1}{2}$ that implies $0\leq a_{1}\leq \frac{1}{2}$. Hence, we find that, for CICA as described in Generic Procedure 1, an optimal solution is to reduce X to *U* and Y to V. This captures all the dependence between the vectors X and Y, which appears to be the most desirable outcome.
  As a final note, we point out that it is conceptually straightforward to evaluate Versions 2 and 3 (conditional expectation) of Generic Procedure 1 in this example, but this would require embedding the considered binary alphabets into the real numbers. This makes it a less satisfying option for the simple example at hand.

## 6. A Gradient Descent Based Implementation

As we discussed above, in our problem, the objective is indeed a convex function of the optimization variables (but the constraint set is not convex). Clearly, this gives hope that gradient-based techniques may lead to interesting solutions. In this section, we examine a first tentative implementation and check it against ground truth for some simple examples.

In theory for convex problems, gradient descent will guarantee convergence to the optimal solution; otherwise, it will guarantee only local convergence. Gradient descent runs in iterative steps, where each step does a local linear approximation and the step size depends on a learning parameter that is α for our problem. In our work, we want to minimize the objective I(W;X,Y) when the constraint I(X;Y|W) is held below a γ−level.

Instead, we apply a variant of gradient descent where we minimize the weighted sum of objective I(W;X,Y) and the constraint I(X;Y|W), which is I(W;X,Y)+λI(X;Y|W). The parameter λ will permit some control on the constraint, thus sweeping all its possible values. We present the algorithm where C(p(w|x,y)) will be a function of p(w|x,y) that will represent I(W;X,Y), and J(p(w|x,y)) will be a function of p(w|x,y) that will represent I(X;Y|W).

The exact computation of the stated update step is presented in the following lemma.

**Lemma** **2** (Computation of the update step)**.** *Let p(x,y) be a fixed distribution, then the updating steps for the gradient descent are*

(59) $$\begin{array}{cc} \frac{\partial C(p(w|x,y)}{\partial p(w|x,y)} & =p\left(x,y\right)\log\frac{p(w|x,y)}{\sum_{x^{\prime },y^{\prime }}p\left(x^{\prime },y^{\prime }\right)p\left(w|x^{\prime },y^{\prime }\right)}, \end{array}$$

(60) $$\begin{array}{cc} \frac{\partial J(p(w|x,y)}{\partial p(w|x,y)} & =p\left(x,y\right)\log\frac{p\left(w|x,y\right)\sum_{x^{\prime },y^{\prime }}p\left(x^{\prime },y^{\prime }\right)p\left(w|x^{\prime },y^{\prime }\right)}{\sum_{x^{\prime \prime }}p\left(w|x^{\prime \prime },y\right)p\left(x^{\prime \prime }|y\right)\sum_{y^{\prime \prime }}p\left(w|x,y^{\prime \prime }\right)p\left(y^{\prime \prime }|x\right)}. \end{array}$$

**Proof.**  Let the function *C* be as defined above

(61) $$\begin{array}{cc} C(p(w|x,y)) & =\sum_{x,y,w}p\left(w|x,y\right)p\left(x,y\right)\log\frac{p(w|xy)}{\sum_{x^{\prime },y^{\prime }}p\left(w|x^{\prime },y^{\prime }\right)p\left(x^{\prime },y^{\prime }\right)}, \end{array}$$

and, in terms of information theoretic terms, the function is C(p(w|x,y))=I(W;X,Y). In addition, C(p(w|x,y)) is a convex function of p(w|x,y), shown in (Theorem 2.7.4 in [29]). Taking the first derivative, we get

$$\begin{array}{cc} \frac{\partial C(p(w|x,y))}{\partial p(w|x,y)} & =p\left(x,y\right)\log\frac{p(w|x,y)}{\sum_{x^{\prime },y^{\prime }}p\left(w|x^{\prime },y^{\prime }\right)p\left(x^{\prime },y^{\prime }\right)}+p\left(w|x,y\right)p\left(x,y\right)\frac{1}{p(w|x,y)} \end{array}$$

(62) $$\begin{array}{cc} \; & \;-\sum_{x^{\prime \prime },y^{\prime \prime }}p\left(w|x^{\prime \prime },y^{\prime \prime }\right)p\left(x^{\prime \prime },y^{\prime \prime }\right)\frac{p(x,y)}{\sum_{x^{\prime },y^{\prime }}p\left(w|x^{\prime },y^{\prime }\right)p\left(x^{\prime },y^{\prime }\right)} \end{array}$$

(63) $$\begin{array}{cc} \; & =p\left(x,y\right)\log\frac{p(w|x,y)}{\sum_{x^{\prime },y^{\prime }}p\left(w|x^{\prime },y^{\prime }\right)p\left(x^{\prime },y^{\prime }\right)}. \end{array}$$

On the other hand, the term I(X;Y|W) can be expressed as

(64) $$\begin{array}{cc} I(X;Y|W) & =I(W;X,Y)-I(W;X)-I(W;Y)+I(X;Y) \end{array}$$

(65) $$\begin{array}{cc} \; & =C(p(w|x,y))-C(p(w|x))-C(p(w|y))+I(X;Y). \end{array}$$

Taking the derivative with respect to p(w|x,y) becomes easier once I(X;Y|W) is written in terms of function *C* and we already know the derivative of *C* from (63). Thus, the derivative would be

(66) $$\begin{array}{cc} \frac{\partial J(p(w|x,y)}{\partial p(w|x,y)} & =\frac{\partial C(p(w|x,y))}{\partial p(w|x,y)}-\frac{\partial C(p(w|x))}{\partial p(w|x,y)}-\frac{\partial C(p(w|y))}{\partial p(w|x,y)} \end{array}$$

(67) $$\begin{array}{cc} \; & =\frac{\partial C(p(w|x,y))}{\partial p(w|x,y)}-\frac{\partial C(p(w|x))}{\partial p(w|x)}\frac{\partial p(w|x)}{\partial p(w|x,y)}-\frac{\partial C(p(w|y))}{\partial p(w|y)}\frac{\partial p(w|y)}{\partial p(w|x,y)}\\ \; & =p\left(x,y\right)\log\frac{p(w|x,y)}{\sum_{x^{\prime },y^{\prime }}p\left(w|x^{\prime },y^{\prime }\right)p\left(x^{\prime },y^{\prime }\right)}-p\left(x\right)\log\frac{p(w|x)}{\sum_{x^{\prime \prime }}p\left(w|x^{\prime \prime }\right)p\left(x^{\prime \prime }\right)}p\left(y|x\right) \end{array}$$

(68) $$\begin{array}{cc} \; & \;-p\left(y\right)\log\frac{p(w|y)}{\sum_{y^{\prime \prime }}p\left(w|y^{\prime \prime }\right)p\left(y^{\prime \prime }\right)}p\left(x|y\right) \end{array}$$

(69) $$\begin{array}{cc} \; & =p\left(x,y\right)\log\frac{p\left(w|x,y\right)\sum_{x^{\prime },y^{\prime }}p\left(x^{\prime },y^{\prime }\right)p\left(w|x^{\prime },y^{\prime }\right)}{\sum_{x^{\prime \prime }}p\left(w|x^{\prime \prime },y\right)p\left(x^{\prime \prime }|y\right)\sum_{y^{\prime \prime }}p\left(w|x,y^{\prime \prime }\right)p\left(y^{\prime \prime }|x\right)}. \end{array}$$

where (67) is an application of the chain rule, and the rest is straightforward computation. □

**Remark** **2.** *In practice, it is useful and computationally cheaper to replace the derivative formulas in Lemma 2 by their standard approximations. That is, the updating step in line 7 of Algorithm 1 is replaced by*

(70) $$\begin{array}{cc} \; & \frac{\partial C(p(w|x,y))}{\partial p(w|x,y)}\approx \frac{C(p(w|x,y)+\Delta )-C(p(w|x,y))}{\Delta }, \end{array}$$

(71) $$\begin{array}{cc} \; & \frac{\partial J(p(w|x,y))}{\partial p(w|x,y)}\approx \frac{J(p(w|x,y)+\Delta )-J(p(w|x,y))}{\Delta }, \end{array}$$

*for a judicious choice of Δ. This is the version that was used for Figure 1b, with $\Delta =10^{-3}$. We point out that, in the general case, the error introduced by this approximation is not bounded.*

**Algorithm 1:** Approximate CICA Algorithm via Gradient Descent

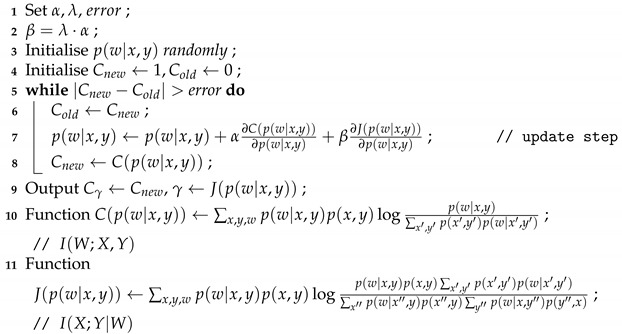



## 7. Extension to More than Two Sources

It is unclear how one would extend CCA to more than two databases. By contrast, for CICA, this extension is conceptually straightforward. For Wyner’s common information, in Definition 1, it suffices to replace the objective in the minimization by $I(X_{1},X_{2},\ldots ,X_{M};W)$ and to keep the constraint of conditional independence. To obtain an interesting algorithm, we now need to relax the constraint of conditional independence. The most natural way is via the conditional version of Watanabe’s total correlation [31], leading to the following definition:

**Definition** **3** (Relaxed Wyner’s Common Information for *M* variables)**.** *For a fixed probability distribution $p(x_{1},x_{2},\ldots ,x_{M}),$ we define*

(72) $$\begin{array}{cc} C_{\gamma }\left(X_{1};X_{2};\ldots ;X_{M}\right) & =\inf I(X_{1},X_{2},\ldots ,X_{M};W) \end{array}$$

*such that $\sum_{i=1}^{M}H\left(X_{i}|W\right)-H\left(X_{1},X_{2},\ldots ,X_{M}|W\right)\leq \gamma ,$ where the infimum is over all probability distributions $p(w,x_{1},x_{2},\ldots ,x_{M})$ with marginal $p(x_{1},x_{2},\ldots ,x_{M}).$*

Not surprisingly, an explicit closed-form solution is difficult to find. One simple case appears below as part of the example presented in Section 7.1, see Lemma 4. By analogy with Lemma 1, we can again state basic properties.

**Lemma** **3.** *$C_{\gamma }\left(X_{1};X_{2};\ldots ;X_{M}\right)$ satisfies the following basic properties:*

*1.*  $C_{\gamma }\left(X_{1};X_{2};\ldots ;X_{M}\right)\geq \frac{1}{M-1}\max\left\{\sum_{i=1}^{M}H\left(X_{i}\right)-H\left(X_{1},X_{2},\ldots ,X_{M}\right)-\gamma ,0\right\}.$
*2.*  *$C_{\gamma }\left(X_{1};X_{2};\ldots ;X_{M}\right)$ is a convex and continuous function of γ for γ≥0.*
*3.*  *If $Z-X_{1}-\left(X_{2},\ldots ,X_{M}\right)$ forms a Markov chain,*
*then $C_{\gamma }\left(\left(X_{1},Z\right);X_{2};\ldots ;X_{M}\right)=C_{\gamma }\left(X_{1};X_{2};\ldots ;X_{M}\right).$*
*4.*  *The cardinality of W may be restricted to $\left|W\right|\leq \prod_{i=1}^{M}\left|X_{i}\right|+1.$*
*5.*  *If $f_{i}\left(\cdot \right)$ are one-to-one functions,*
*then $C_{\gamma }\left(f_{1}\left(X_{1}\right);f_{2}\left(X_{2}\right);\ldots ;f_{M}\left(X_{M}\right)\right)=C_{\gamma }\left(X_{1};X_{2};\ldots ;X_{M}\right).$*
*6.*  *For discrete X, we have $C_{\gamma }\left(X;X;\ldots ;X\right)=\max\left\{H\left(X\right)-\frac{\gamma }{M-1},0\right\}.$*

Proofs for these basic properties can be found in Appendix C.

Leveraging Definition 3, it is conceptually straightforward to extend CICA (that is, Generic Procedure 1) to the case of *M* databases as follows. For completeness, we include an explicit statement of the resulting procedure.

**Generic Procedure 2**  (CICA with multiple sources)**.**

*1.*  Select a real number γ, where $0\leq \gamma \leq \sum_{i=1}^{M}H\left(X_{i}\right)-H\left(X_{1},X_{2},\ldots ,X_{M}\right).$ This is the compression level: A low value of γ represents low compression, and, thus, many components are retained. A high value of γ represents high compression, and, thus, only a small number of components are retained.
*2.*  *Solving the relaxed Wyner’s common information problem, -4.6cm0cm*
  (73) $$\begin{array}{c} \min_{p(w|x_{1},x_{2},\ldots ,x_{M})}I\left(X_{1},X_{2},\ldots ,X_{M};W\right)\;such\;that\;\sum_{i=1}^{M}H\left(X_{i}|W\right)-H\left(X_{1},X_{2},\ldots ,X_{M}|W\right)\leq \gamma , \end{array}$$
  leading to an associated conditional distribution $p_{\gamma }\left(w|x_{1},x_{2},\ldots ,x_{M}\right).$
*3.*  *Using the conditional distribution $p_{\gamma }\left(w|x_{1},x_{2},\ldots ,x_{M}\right)$ found in Step 2, the dimension-reduced data sets can now be found via one of the following three variants:*
  *(a)*  *Version 1: MAP (maximum a posteriori):*
    (74) $$\begin{array}{cc} u_{i}\left(x_{i}\right) & =\arg\max_{w}p_{\gamma }\left(w|x_{i}\right), \end{array}$$
    for i=1,2,…,M.
  *(b)*  *Version 2: Conditional Expectation:*
    (75) $$\begin{array}{cc} u_{i}\left(x_{i}\right) & =E[W|X_{i}=x_{i}] \end{array}$$
    for i=1,2,…,M.
  *(c)*  *Version 3: Marginal Integration:*
    (76) $$\begin{array}{cc} u_{i}\left(x_{i}\right) & =\int_{x_{1},\ldots ,x_{i-1},x_{i+1},\ldots ,x_{M}}p\left(x_{1},\ldots ,x_{i-1},x_{i+1},\ldots ,x_{M}\right)\\ \; & E\left[W|X_{1}=x_{1},\ldots ,X_{M}=x_{M}\right]dx_{1}\cdots dx_{i-1}dx_{i+1}\cdots dx_{M} \end{array}$$
    for i=1,2,…,M.

Clearly, Generic Procedure 2 closely mirrors Generic Procedure 1. The key difference is that there is no direct analog of Theorem 2. This is no surprise since it is unclear how CCA would be extended to beyond the case of two sources. Nonetheless, it would be very interesting to explore what Generic Procedure 2 boils down to in the special case when all vectors are jointly Gaussian. At the current time, this is unknown. In fact, the explicit solution to the optimization problem in Definition 3 is presently an open problem.

Instead, we illustrate the promise of Generic Procedure 2 via a simple binary example in the next section. The example mirrors some of the basic properties of the example tackled in Section 5.

### 7.1. A Binary Example with Three Sources

In this section, we develop an example with three sources that borrows some of the ideas from the example discussed in Section 5. In a sense, the present example is even more illustrative because, in it, *any* two of the original vectors $X_{1},X_{2},$ and $X_{3},$ are (pairwise) independent. Therefore, any method based on pairwise measures, including CCA and maximal correlation, would not identify any commonality at all. Specifically, we consider the following simple statistical model:

(77) $$\begin{array}{c} X_{1}=\left(\begin{array}{c} U\\ Z_{1} \end{array}\right),X_{2}=\left(\begin{array}{c} V\\ Z_{2} \end{array}\right),X_{3}=\left(\begin{array}{c} U⊕V\\ Z_{3} \end{array}\right), \end{array}$$

where $U,V,Z_{1},Z_{2},Z_{3}$ are independent uniform binary variables and ⊕ denotes modulo-2 addition. We observe that, amongst these three vectors, any pair is independent. This implies, for example, that any correlation-based technique (including maximal correlation) will not identify any relevant features, since correlation is a pairwise measure. By contrast, we can show that one output of Algorithm 2 is indeed to select W=(U,V), for γ=0. Thus, the algorithm would reduce each of the three vectors to their first component, which is the intuitively pleasing answer in this case. By going through the steps of the Generic Procedure 2, for γ=0, where the the joint distribution satisfies

(78) $$\begin{array}{c} p\left(u,v,u⊕v,z_{1},z_{2},z_{3}\right)=p\left(u,v,u⊕v\right)p\left(z_{1}\right)p\left(z_{2}\right)p\left(z_{3}\right) \end{array}$$

we have that

(79) $$\begin{array}{cc} C(X_{1};X_{2};X_{3}) & =C(U,Z_{1};V,Z_{2};U⊕V,Z_{3}) \end{array}$$

(80) $$\begin{array}{cc} \; & =C(U;V,Z_{2};U⊕V,Z_{3}) \end{array}$$

(81) $$\begin{array}{cc} \; & =C(U;V;U⊕V,Z_{3}) \end{array}$$

(82) $$\begin{array}{cc} \; & =C(U;V;U⊕V) \end{array}$$

where we use Lemma 3, Item 3, together with the Markov chain $Z_{1}-U-\left(Z_{2},V,Z_{3},U⊕V\right)$ that follows from (78) to prove step (80). Similarly, the Markov chain $Z_{2}-V-\left(U,Z_{3},U⊕V\right)$ proves step (81) by making use of Lemma 3, Item 3. A similar argument is used for the last step (82). Managing to compute C(U;V;U⊕V) is equivalent to computing $C(X_{1};X_{2};X_{3})$, and we demonstrate how to compute it in the next part.

**Lemma** **4.** *Let U,V be independent uniform binary variables and ⊕ denotes modulo-2 addition. Then, the optimal solution to*

(83) $$\begin{array}{c} C_{\gamma =0}\left(U;V;U⊕V\right)=\underset{W:H(U|W)+H(V|W)+H(U⊕V|W)-H(U,V,U⊕V|W)=0}{\inf}I\left(W;U,V,U⊕V\right) \end{array}$$

*is W=(U,V), where the expression evaluates to two.*

The proof is given in Appendix D. If we apply Version 1 of Step 3 of Generic Procedure 2, we obtain

(84) $$\begin{array}{c} \arg\max_{w}p_{\gamma =0}\left(w|x_{1}\right)=\left\{\left(u,0\right),\left(u,1\right)\right\}, \end{array}$$

that is, in this case, the maximizer is not unique. However, as we observe that the set of maximizers is a deterministic function of *u* alone, it is natural to reduce as follows:

(85) $$\begin{array}{cc} u_{1}\left(x_{1}\right) & =u. \end{array}$$

By the same token, we can reduce

(86) $$\begin{array}{cc} u_{2}\left(x_{2}\right) & =v, \end{array}$$

(87) $$\begin{array}{cc} u_{3}\left(x_{3}\right) & =u⊕v. \end{array}$$

In this example, it is clear that this indeed extracts all of the dependency there is between our three sources, and, thus, is the correct answer.

As pointed out above, in this simple example, any pair of the random vectors $X_{1},X_{2},$ and $X_{3}$ are (pairwise) independent, which implies that the classic tools based on pairwise measures (CCA, maximal correlation) cannot identify any commonality between $X_{1},X_{2},$ and $X_{3}.$

## 8. Conclusions and Future Work

We introduce a novel two-step procedure that we refer to as CICA. The first step consists of an information minimization problem related to Wyner’s common information, while the second can be thought of as a type of back-projection. We prove that, in the special case of Gaussian statistics, this two-step procedure precisely extracts the CCA components. A free parameter γ in CICA permits selecting the number of CCA components that are being extracted. In this sense, the paper establishes a novel rigorous connection between CCA and information measures. A number of simple examples are presented. It is also shown how to extend the novel algorithm to more than two sources.

Future work includes a more in-depth study and consideration to assess the practical promise of this novel algorithm. This will also require moving beyond the current setting where it was assumed that the probability distribution of the data at hand was provided directly. Instead, this distribution has to be estimated from data, and one needs to understand what limitations this additional constraint will end up imposing.

## Data Availability

The dataset is available from the corresponding author on request.

## Abbreviations

The following abbreviations are used in this manuscript:

CCA	Canonical correlation analysis
ACE	Alternating conditional expectation
ICA	Independent component analysis
CICA	Common information component analysis

## Appendix A. Proof of Lemma 1

For Item 5, on the one hand, we have

(A1) $$\begin{array}{cc} C_{\gamma }\left(\left(X,Z\right);Y\right) & =\underset{p(w|x,y,z):I(X,Z;Y|W)\leq \gamma }{\inf}I\left(X,Z,Y;W\right) \end{array}$$

(A2) $$\begin{array}{cc} \; & \leq \underset{p(w|x,y):I(X;Y|W)+I(Z;Y|W,X)\leq \gamma }{\inf}I\left(X,Y;W\right)+I\left(Z;W|X,Y\right) \end{array}$$

(A3) $$\begin{array}{cc} \; & =C_{\gamma }\left(X;Y\right) \end{array}$$

where, in Equation (A2), we add the constraint that conditioned on (X,Y),*W* is selected to be *independent* of Z, which cannot reduce the value of the infimum. That is, for such a choice of W, we have the Markov chain Z−(X,Y)−W, thus I(Z;W|X,Y)=0. Furthermore, observe that the factorization p(x,y,z,w)=p(x,y)p(z|x)p(w|x,y) also implies the factorization p(x,y,z,w)=p(x,w)p(z|x)p(y|w,x). Hence, we also have the Markov chain Z−(W,X)−Y; thus, I(Z;Y|W,X)=0, which thus established the last step. Conversely, observe that

(A4) $$\begin{array}{cc} C_{\gamma }\left(\left(X,Z\right);Y\right) & =\underset{p(w|x,y,z):I(X,Z;Y|W)\leq \gamma }{\inf}I\left(X,Y,Z;W\right) \end{array}$$

(A5) $$\begin{array}{cc} \; & \geq \underset{p(w|x,y):I(X;Y|W)\leq \gamma }{\inf}I\left(X,Y;W\right)+\underset{p(w|x,y,z):I(X,Z;Y|W)\leq \gamma }{\inf}I\left(Z;W|X,Y\right) \end{array}$$

(A6) $$\begin{array}{cc} \; & \geq C_{\gamma }\left(X;Y\right) \end{array}$$

where (A5) follows from the fact that the infimum of the sum is lower bounded by the sum of the infimums and the fact that relaxing constraints cannot increase the value of the infimum, and (A6) follows from non-negativity of the second term.

Item 6 is a standard cardinality bound, following from the arguments in [32]. For the context at hand, see also Theorem 1 in (p. 6396, [33]). Item 7 follows because all involved mutual information terms are invariant to one-to-one transforms. For Item 8), note that we can express $C_{\gamma }\left(X;X\right)=H\left(X\right)-\max_{p(w|x):H(X|W)\leq \gamma }H\left(X|W\right),$ which directly gives the result.

## Appendix B. A Brief Review of Canonical Correlation Analysis (CCA)

A brief review of CCA [1] is presented. Let X and Y be zero-mean real-valued random vectors with covariance matrices $K_{X}$ and $K_{Y},$ respectively. Moreover, let $K_{\mathrm{XY}}=E\left[XY^{H}\right].$ We first apply the change of basis as in (24) and (25). CCA seeks to find vectors u and v such as to maximize the correlation between $u^{H}\hat{X}$ and $v^{H}\hat{Y},$ that is,

(A7) $$\begin{array}{c} \max_{u,v}\frac{E[u^{H}\hat{X}{\hat{Y}}^{H}v]}{\sqrt{E[|u^{H}\hat{X}{|}^{2}]}\sqrt{E[|v^{H}\hat{Y}{|}^{2}]}}, \end{array}$$

which can be rewritten as

(A8) $$\begin{array}{c} \max_{u,v}\frac{u^{H}K_{\hat{X}\hat{Y}}v}{∥u∥\;\;∥v∥}, \end{array}$$

where

(A9) $$\begin{array}{cc} K_{\hat{X}\hat{Y}} & =K_{X}^{-1/2}K_{\mathrm{XY}}K_{Y}^{-1/2}. \end{array}$$

Note that this expression is *invariant* to arbitrary (separate) scaling of u and v. To obtain a unique solution, we could choose to impose that both vectors be unit vectors,

(A10) $$\begin{array}{c} \max_{u,v:∥u∥=∥v∥=1}u^{H}K_{\hat{X}\hat{Y}}v. \end{array}$$

From Cauchy–Schwarz, for a fixed u, the maximizing (unit-norm) v is given by

(A11) $$\begin{array}{cc} v & =\frac{K_{\hat{X}\hat{Y}}^{H}u}{∥K_{\hat{X}\hat{Y}}^{H}u∥}, \end{array}$$

or, equivalently, for a fixed v, the maximizing (unit-norm) u is given by

(A12) $$\begin{array}{cc} u & =\frac{K_{\hat{X}\hat{Y}}v}{∥K_{\hat{X}\hat{Y}}v∥}. \end{array}$$

Plugging in the latter, we obtain

(A13) $$\begin{array}{c} \max_{v:∥v∥=1}\frac{v^{H}K_{\hat{X}\hat{Y}}^{H}K_{\hat{X}\hat{Y}}v}{∥K_{\hat{X}\hat{Y}}v∥}, \end{array}$$

or, dividing through,

(A14) $$\begin{array}{c} \max_{v:∥v∥=1}∥K_{\hat{X}\hat{Y}}v∥. \end{array}$$

The solution to this problem is well known: v is the right singular vector corresponding to the largest singular vector of the matrix $K_{\hat{X}\hat{Y}}=K_{X}^{-1/2}K_{\mathrm{XY}}K_{Y}^{-1/2}.$ Evidently, u is the corresponding left singular vector. Restarting again from Equation (A7), but restricting to vectors that are orthogonal to the optimal choices of the first round leads to the second CCA components, and so on.

## Appendix C. Proof of Lemma 3

For item 1, we proceed as follows

(A15) $$\begin{array}{cc} C_{\gamma }\left(X_{1};X_{2};\cdots ;X_{M}\right) & =\underset{W:H\left(X_{1}|W\right)+H\left(X_{2}|W\right)+\cdots +H\left(X_{M}|W\right)-H\left(X_{1},X_{2},\cdots ,X_{M}|W\right)\leq \gamma }{\inf}\;I\left(W;X_{1},X_{2},\cdots ,X_{M}\right) \end{array}$$

(A16) $$\begin{array}{cc} \; & \geq \underset{W}{\inf}L\left(\lambda ,p\left(w|x_{1},x_{2},\cdots ,x_{M}\right)\right) \end{array}$$

where we used weak duality for λ≥0 and $L(\lambda ,p\left(w|x_{1},x_{2},\cdots ,x_{M}\right)$ is

(A17) $$\begin{array}{cc} L(\lambda ,p\left(w|x_{1},x_{2},\cdots ,x_{M}\right) & :=I\left(W;X_{1},X_{2},\cdots ,X_{M}\right)+\lambda [H\left(X_{1}|W\right)\\ \; & \;\;+H\left(X_{2}|W\right)+\cdots +H\left(X_{M}|W\right)-H\left(X_{1},X_{2},\cdots ,X_{M}|W\right)-\gamma ]. \end{array}$$

By setting $\lambda =\frac{1}{M-1}$, we obtain

(A18) $$\begin{array}{cc} \; & L(\frac{1}{M-1},p\left(w|x_{1},x_{2},\cdots ,x_{M}\right))\\ \; & \;=\frac{M}{M-1}I\left(W;X_{1},X_{2},\cdots ,X_{M}\right)-\frac{1}{M-1}\left[I\left(W;X_{1}\right)+I\left(W;X_{2}\right)+\cdots +I\left(W;X_{M}\right)\right] \end{array}$$

(A19) $$\begin{array}{cc} \; & \;+\frac{1}{M-1}\left[H\left(X_{1}\right)+H\left(X_{2}\right)+\cdots +H\left(X_{M}\right)-H\left(X_{1},X_{2},\cdots ,X_{M}\right)-\gamma \right]\\ \; & \;=\frac{1}{M-1}[I\left(W;X_{2},\cdots ,X_{M}|X_{1}\right)+\cdots +\frac{1}{M-1}[I\left(W;X_{1},\cdots ,X_{M-1}|X_{M}\right) \end{array}$$

(A20) $$\begin{array}{cc} \; & \;+\frac{1}{M-1}\left[H\left(X_{1}\right)+H\left(X_{2}\right)+\cdots +H\left(X_{M}\right)-H\left(X_{1},X_{2},\cdots ,X_{M}\right)-\gamma \right], \end{array}$$

where the infimum of $L(\frac{1}{M-1},p\left(w|x_{1},x_{2},\cdots ,x_{M}\right))$ in (A20) is attained for the trivial random variable *W*, thus $C_{\gamma }\left(X_{1};X_{2};\cdots ;X_{M}\right)\geq \frac{1}{M-1}\left[H\left(X_{1}\right)+H\left(X_{2}\right)+\cdots +H\left(X_{M}\right)-H\left(X_{1},X_{2},\cdots ,X_{M}\right)-\gamma \right]$. Item 2 follows from a similar argument as in (Corollary 4.5 in [23]). For item 3, we start by showing both sides of the inequality that will result in equality. One side of the inequality is shown below:

(A21) $$\begin{array}{cc} \; & C_{\gamma }\left(X_{1},Z;X_{2};\cdots ;X_{M}\right) \end{array}$$

(A22) $$\begin{array}{cc} \; & \;=\underset{\begin{array}{c} W:H\left(X_{1},Z|W\right)+H\left(X_{2}|W\right)+\cdots +H\left(X_{M}|W\right)-H\left(X_{1},Z,X_{2},\cdots ,X_{M}|W\right)\leq \gamma \end{array}}{\inf}\;I\left(W;X_{1},Z,X_{2},\cdots ,X_{M}\right) \end{array}$$

(A23) $$\begin{array}{cc} \; & \;=\underset{\begin{array}{c} W:H\left(X_{1}|W\right)+H\left(X_{2}|W\right)+\cdots +H\left(X_{M}|W\right)-H\left(X_{1},X_{2},\cdots ,X_{M}|W\right)\\ +I(Z;X_{2},\cdots ,X_{M}|X_{1},W)\leq \gamma \end{array}}{\inf}\;I\left(W;X_{1},X_{2},\cdots ,X_{M}\right)+I\left(W;Z|X_{1},X_{2},\cdots ,X_{M}\right) \end{array}$$

(A24) $$\begin{array}{cc} \; & \;\leq C_{\gamma }\left(X_{1};X_{2};\cdots ;X_{M}\right) \end{array}$$

where the last inequality follows by restricting the possible set of *W*, such that *W* and *Z* are conditionally independent given $\left(X_{1},X_{2},\cdots ,X_{M}\right)$,

(A25) $$\begin{array}{c} I(Z;W|X_{1},X_{2},\cdots ,X_{M})=0. \end{array}$$

From the statement of the lemma, we have $Z-X_{1}-\left(X_{2},\cdots ,X_{M}\right)$,

(A26) $$\begin{array}{c} I(Z;X_{2},\cdots ,X_{M}|X_{1})=0. \end{array}$$

By adding (A25) and (A26), we get $I(Z;W,X_{2},\cdots ,X_{M}|X_{1})=0$. This implies that we have $I(Z;X_{2},\cdots ,X_{M}|X_{1},W)=0$, which appears in the constraint of (A23). For the other part of the inequality we proceed as follows:

(A27) $$\begin{array}{cc} \; & C_{\gamma }\left(X_{1},Z;X_{2};\cdots ;X_{M}\right) \end{array}$$

(A28) $$\begin{array}{cc} \; & \;=\underset{\begin{array}{c} W:H\left(X_{1}|W\right)+H\left(X_{2}|W\right)+\cdots +H\left(X_{M}|W\right)-H\left(X_{1},X_{2},\cdots ,X_{M}|W\right)\\ +I(Z;X_{2},\cdots ,X_{M}|X_{1},W)\leq \gamma \end{array}}{\inf}\;I\left(W;X_{1},X_{2},\cdots ,X_{M}\right)+I\left(W;Z|X_{1},X_{2},\cdots ,X_{M}\right) \end{array}$$

(A29) $$\begin{array}{cc} \; & \;\geq C_{\gamma }\left(X_{1};X_{2};\cdots ;X_{M}\right), \end{array}$$

where the last part follows by relaxing the constraint set as $I(Z;X_{2},\cdots ,X_{M}|X_{1},W)\geq 0$ and, by further bounding the terms in the objective, $I(W;Z|X_{1},X_{2},\cdots ,X_{M})\geq 0$.

Item 4 is a standard cardinality bound, following from a similar argument in [32]. Item 5 follows because all involved mutual information terms are invariant to one-to-one transforms. For item 6, we apply the definition of relaxed Wyner’s common information for *M* variables, and we have

(A30) $$\begin{array}{cc} C_{\gamma }\left(X;X;\cdots ;X\right) & =\underset{W:(M-1)H(X|W)\leq \gamma }{\inf}I\left(X;W\right) \end{array}$$

(A31) $$\begin{array}{cc} \; & =H\left(X\right)-\underset{W:(M-1)H(X|W)\leq \gamma }{\sup}H\left(X|W\right) \end{array}$$

(A32) $$\begin{array}{cc} \; & =H\left(X\right)-\frac{\gamma }{M-1}. \end{array}$$

## Appendix D. Proof of Lemma 4

An upper bound to the problem is to pick W=(U,V), thus

(A33) $$\begin{array}{c} C(U;V;U⊕V)\leq H(U,V,U⊕V)=2. \end{array}$$

Another equivalent way of writing the problem is by splitting the constraint into two constraints, as we already know that the constraint cannot be smaller than zero, so it has to be exactly zero, and it can be written in the following way:

(A34) $$\begin{array}{c} C\left(U;V;U⊕V\right)=\underset{\begin{array}{c} W:I(U⊕V;U,V|W)=0\\ I(U;V|W)=0 \end{array}}{\inf}I\left(W;U,V,U⊕V\right). \end{array}$$

By using weak duality for λ≥0, a lower bound to the problem would be the following

(A35) $$\begin{array}{cc} C(U;V;U⊕V) & \geq \underset{W:U-W-V}{\inf}I\left(W;U,V,U⊕V\right)+\lambda [H\left(U,V|W\right)+H\left(U⊕V|W\right)\\ \; & \;\;-H(U,V,U⊕V|W)]. \end{array}$$

By further using the constraint U−W−V, the above expression can be written as

(A36) $$\begin{array}{cc} C(U;V;U⊕V) & \geq \underset{W:U-W-V}{\inf}I\left(W;U,V,U⊕V\right)+\lambda [H\left(U|W\right)+H\left(V|W\right)+H\left(U⊕V|W\right)\\ \; & \;\;-H(U,V,U⊕V|W)] \end{array}$$

(A37) $$\begin{array}{cc} \; & =H\left(U,V,U⊕V\right)+\underset{W:U-W-V}{\inf}\lambda \left[H\left(U|W\right)+H\left(V|W\right)+H\left(U⊕V|W\right)\right]\\ \; & \;\;-(1+\lambda )H(U,V,U⊕V|W) \end{array}$$

(A38) $$\begin{array}{cc} \; & \geq H\left(U,V,U⊕V\right)+\underset{\tilde{U},\tilde{V}}{\inf}\underset{\begin{array}{c} W:\tilde{U}-W-\tilde{V}\\ \tilde{U}⊕\tilde{V}-\left(\tilde{U},\tilde{V}\right)-W \end{array}}{\inf}\;\lambda \left[H\left(\tilde{U}|W\right)+H\left(\tilde{V}|W\right)+H\left(\tilde{U}⊕\tilde{V}|W\right)\right]\\ \; & \;\;-\left(1+\lambda \right)H\left(\tilde{U},\tilde{V},\tilde{U}⊕\tilde{V}|W\right) \end{array}$$

(A39) $$\begin{array}{cc} \; & =2+\underset{\tilde{U},\tilde{V}}{\inf}\underset{L{\left[\lambda H\left(\tilde{U}⊕\tilde{V}\right)-H\left(\tilde{U}\right)-H\left(\tilde{V}\right)\right]}_{p_{\tilde{U}}p_{\tilde{V}}}}{\underset{︸}{\underset{\begin{array}{c} W:\tilde{U}-W-\tilde{V}\\ \tilde{U}⊕\tilde{V}-\left(\tilde{U},\tilde{V}\right)-W \end{array}}{\inf}\lambda H\left(\tilde{U}⊕\tilde{V}|W\right)-H\left(\tilde{U}|W\right)-H\left(\tilde{V}|W\right)}} \end{array}$$

where (A38) is a consequence of allowing a minimization (if minimum exists) over binary random variables $\tilde{U},\tilde{V}$ and the rest of equalities is straightforward manipulation. The last equation is in terms of the lower convex envelope with respect to the distribution $p_{\tilde{U}}p_{\tilde{V}}$. The aim is to search for the tightest bound over λ by studying the lower convex envelope with respect to $p_{\tilde{U}}p_{\tilde{V}}$, which, for binary $\tilde{U},\tilde{V}$, can be simplified into

(A40) $$\begin{array}{c} L{\left[\lambda H\left(\tilde{U}⊕\tilde{V}\right)-H\left(\tilde{U}\right)-H\left(\tilde{V}\right)\right]}_{p_{\tilde{U}}p_{\tilde{V}}}=L{\left[\lambda h_{b}\left(\alpha \beta +\left(1-\alpha \right)\left(1-\beta \right)\right)-h_{b}\left(\alpha \right)-h_{b}\left(\beta \right)\right]}_{\alpha ,\beta } \end{array}$$

and the latter function is a lower convex envelope with respect to 0≤α,β≤1. Note that (A40) is a continuous function of α,β, so a first order and a second order differentiation will be enough to compute the lower convex envelope. As a result for λ≥2, the lower convex envelope of the right-hand side of (A40) is just zero, thus completing the proof.
